# Tenascin-C and fibronectin in normal esophageal mucosa, Barrett’s esophagus, dysplasia and adenocarcinoma

**DOI:** 10.18632/oncotarget.19196

**Published:** 2017-07-12

**Authors:** Joni Leppänen, Sara Bogdanoff, Petri P. Lehenkari, Juha Saarnio, Joonas H. Kauppila, Tuomo J. Karttunen, Heikki Huhta, Olli Helminen

**Affiliations:** ^1^ Cancer and Translational Medicine Research Unit, Medical Research Center Oulu, University of Oulu and Oulu University Hospital, Oulu, Finland; ^2^ Department of Molecular Medicine and Surgery, Karolinska Institutet and Karolinska University Hospital, Stockholm, Sweden

**Keywords:** esophageal adenocarcinoma, Barrett’s esophagus, tenascin-C, fibronectin, extracellular matrix

## Abstract

**Background:**

Tenascin-C and fibronectin are adhesive glycoproteins modulating the structure of the extracellular matrix and cellular functions. Their expression and function in esophageal adenocarcinoma is poorly known. The aim of this study was to evaluate the expression of tenascin-C and fibronectin in esophageal adenocarcinoma and its precursor stages.

**Results:**

Stromal tenascin-C and fibronectin expression were found in all evaluated lesion types. Expression of both molecules increased from gastric metaplasia towards adenocarcinoma (p<0.05). In carcinomas, tenascin-C expression in the bulk was associated with T-stage (p=0.006), presence of lymph node (p=0.004) and distant organ metastases (p=0.007). Abundant tenascin-C expression associated with poor survival (p=0.034) in univariate analysis. Fibronectin expression associated to T-stage (p=0.030). Expression of tenascin-C or fibronectin in the tumor invasive front was not associated to clinicopathological variables or survival. No significant correlation with tumor/stroma percentage, cancer-associated fibroblasts or mean vascular density was observed with either tenascin-C or fibronectin.

**Methods:**

Tenascin-C and fibronectin were stained immunohistochemically and assessed in esophageal specimens from patients with esophageal adenocarcinoma (n=90) or dysplasia (n=30). Structures and lesion were evaluated including normal esophagus (n=77), gastric (n=61) or intestinal (n=51) metaplasia without dysplasia, and low-grade (n=42) or high-grade (n=34) dysplasia, and esophageal adenocarcinoma (n=90). In carcinomas, both bulk and invasive front were separately evaluated. In addition, tumor/stroma percentage, cancer-associated fibroblasts and mean vascular density were evaluated.

**Conclusions:**

Tenascin-C and fibronectin are upregulated in esophageal adenocarcinoma when compared to Barrett’s esophagus and dysplasia. Increased tenascin-C expression is associated with metastasis and poor prognosis in esophageal adenocarcinoma.

## INTRODUCTION

The incidence of esophageal adenocarcinoma has been increasing rapidly in the Western World during the last decades. Its relative rareness and aggressive nature makes it a disease with late diagnosis and poor survival rate [1]. To improve the prognosis of esophageal adenocarcinoma, early identification of premalignant lesions would be beneficial.

Tenascins are a family of large adhesive of glycoproteins of the extracellular matrix that affect tissue elasticity and architecture and cellular responses [2]. A member of this family, tenascin-C, has an essential role in morphogenesis of blood vessels during embryonic development but its distribution in adult tissues is typically limited. However, re-expression of tenascin-C occurs in injured tissues and in pathological remodeling in various diseases, such as cancer [2]. Increased expression of tenascin-C has been detected in several types of benign and malignant neoplasms, and is found to associate to the invasive and metastatic potential of malignant tumors, for example esophageal squamous cell carcinoma and colorectal cancer [3, 4]. A single study has reported no expression of tenascin-C in 6 patients with Barrett’s esophagus and upregulation of tenascin-C in 15 patients with esophageal adenocarcinoma, showing association to poor tumor differentiation [5].

Fibronectin is another high-molecular-weight adhesive glycoprotein of the extracellular matrix with elevated expression mainly in embryonic tissues, wound healing, inflammation and a variety of tumors, suggesting a role in tissue remodeling [2]. Fibronectin is believed to promote proliferation and survival of tumor cells, and to have a role in tumor invasion and metastases in lung, breast and colorectal cancer [6–8]. No published information on tenascin-C or fibronectin in esophageal adenocarcinoma or its precursor stages could be found in larger patient cohorts.

The aim of the study was to determine the expression of tenascin-C and fibronectin in esophageal adenocarcinoma and its precursor stages, and to evaluate if the molecules could be used as prognostic factors or as biomarkers of premalignant lesions.

## RESULTS

**Figure 1:**
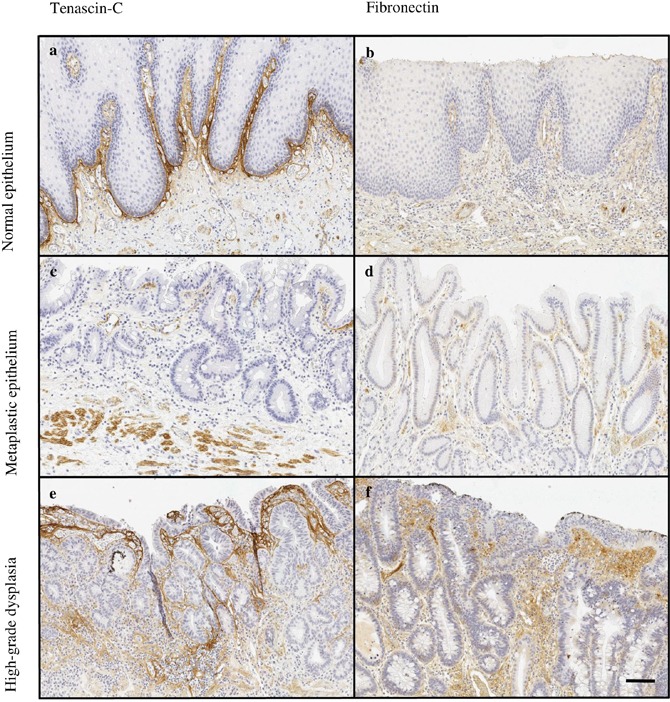
Typical expressions patterns of tenascin-C **(a, c, e)** and fibronectin **(b, d, f)** in normal esophageal mucosa (a, b) and premalignant lesions of adenocarcinoma, including intestinal (c) and gastric (d) metaplasia and high-grade (e, f) dysplasia. Magnification 10x, scale bar 100 μm.

**Figure 2:**
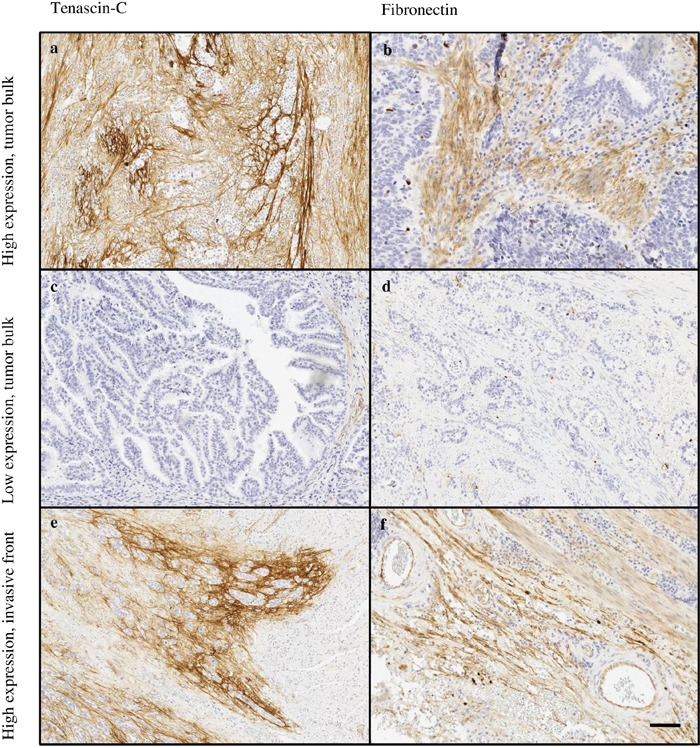
Typical expression patterns of tenascin-C **(a, c, e)** and fibronectin **(b, d, f)** in esophageal adenocarcinoma. Highly variable immunostaining stromal density was observed in tumor stroma of both tenascin-C (a, c) and fibronectin (b, d). The tumor’s invasive front showed high expression of both molecules (e, f). Magnification 10x, scale bar 100 μm.

Both tenascin-C and fibronectin were distinctly expressed in the lamina propria of normal esophageal mucosa covered by squamous epithelium and in the lamina or stroma of all evaluated lesion types, including intestinal and gastric metaplasia, low- and high-grade dysplasia and adenocarcinoma (Figures 1 and 2). The expression of both molecules increased significantly from gastric metaplasia towards adenocarcinoma. Generally expression of tenascin-C was higher than that of fibronectin. Stromal expression levels in the evaluated lesions are summarized in Table 1. For both molecules, cytoplasmic expression in epithelial cells was very rare in normal, metaplastic and neoplastic epithelia and did not associate to any of the clinicopathological variables. Epithelial tenascin-C expression occurred in 16/354 and that for fibronectin in 7/354 of the evaluated lesions, and there was no association with the lesion type (data not shown).

**Table 1:** Stromal expression of tenascin-C and fibronectin in normal esophageal mucosa and in different esophageal lesions

Tenascin-C	Stroma			Invasive front (carcinoma)		
	Mean	95% CI	Statistical significance	Mean	95% CI	Statistical significance
Normal mucosa	1.8	1.6-2.0				
Gastric metaplasia	0.9	0.7-1.1	a			
Intestinal metaplasia	1.0	0.7-1.3	a			
Low-grade dysplasia	1.1	0.8-1.4	a			
High-grade dysplasia	1.6	1.2-2.0	b			
Adenocarcinoma	1.9	1.7-2.2	bcd	2.3	2.0-2.6	f
**Fibronectin** Normal mucosa	0.8	0.7-1.0				
Gastric metaplasia	0.6	0.5-0.8				
Intestinal metaplasia	0.8	0.6-1.0				
Low-grade dysplasia	0.8	0.5-1.0				
High-grade dysplasia	1.0	0.7-1.3				
Adenocarcinoma	1.5	1.3-1.7	abcde	2.1	1.8-2.3	f

Density of stromal expression was assessed with a five point scale from negative (0) to very high density (4). Values are presented as mean and 95% confidence interval (CI). Statistical testing was performed with One way ANOVA with Tukey in post hoc analysis and paired sample t-test.

a compared to normal mucosa, p<0.05.

b compared to gastric metaplasia, p<0.05.

c compared to intestinal metaplasia, p<0.05.

d compared to low-grade dysplasia, p<0.05.

e compared to high-grade dysplasia, p<0.05.

f comparison between intralesional adenocarcinoma stroma and invasive front.

Most typically, in both types of metaplasia and dysplasia stromal expression of tenascin-C and fibronectin was mainly restricted to lamina propria and the underlying connective tissue (Figure 1). In adenocarcinoma the stromal expression of both molecules was more diffuse and the molecules could be identified not only in the middle of the tumor but also in the tumor surrounding stroma (Figure 2).

### Tenascin and fibronectin in normal esophageal mucosa

Expression of tenascin-C in normal mucosa was present in the subepithelial layer and occasionally around the glands (Figure 1). Stromal expression of tenascin-C in normal mucosa was higher than in Barrett’s esophagus or dysplasia, and nearly similar compared to adenocarcinoma (Figures 1 and 2; Table 1).

Fibronectin was only scantily expressed in normal mucosa with locations similar to those of tenascin-C (Figure 1). Expression levels were similar to that in gastric and intestinal metaplasia and in low- and high-grade dysplasia. Statistically significant difference was observed only in comparison between normal epithelium and adenocarcinoma (Table 1).

### Tenascin-C and fibronectin in Barrett’s esophagus and dysplasia

The expression of both of the molecules was lowest in gastric metaplasia and increased gradually towards high-grade dysplasia. The expression was localized in lamina propria and was also seen in the mucosa (Figure 1). The increase from metaplasia to dysplasia was greater with tenascin-C, although only the difference between gastric metaplasia and high-grade dysplasia was statistically significant. No statistically significant differences in fibronectin expression were observed between the premalignant lesions (Table 1).

### Tenascin-C and fibronectin in esophageal adenocarcinoma

Stromal expression of both tenascin-C and fibronectin was highest in adenocarcinoma (Figure 2). The expression of tenascin-C was statistically significantly elevated when compared to all other lesion types, except normal epithelium. Fibronectin expression in adenocarcinoma showed distinct increase when compared to all other lesion types.

Expression in the invasive front was higher than in tumor bulk stroma mean difference of tenascin-C expression being 0.7 (95% CI 0.3-1.1; p<0.001) and that of fibronectin 0.7 (95% CI 0.4-1.0; p<0.001; Table 1).

### Relation with clinicopathological variables and cancer survival

**Figure 3:**
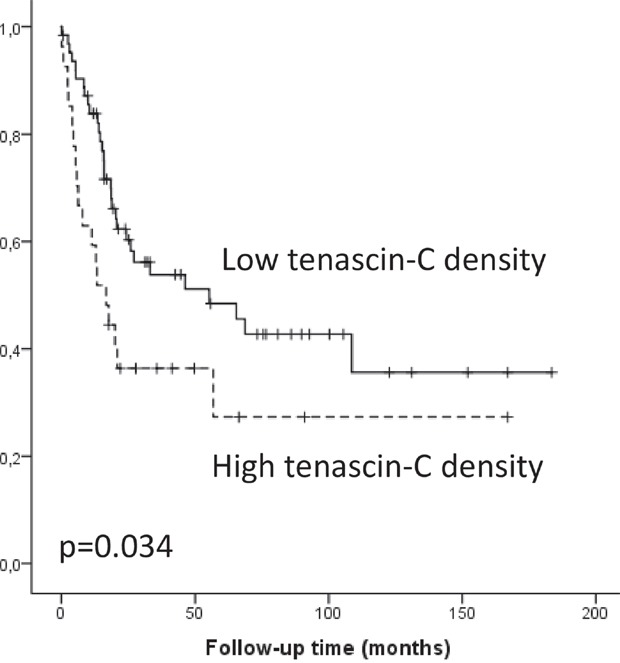
Kaplan-Meier survival curve showing esophageal adenocarcinoma survival divided by low and high (≥3) tenascin-C expression density

Tenascin-C expression in the tumor bulk stroma was associated with several clinocopathological variables (Table 2) including high T-class (p=0.006), lymph node (p=0.004) and organ metastases (p=0.007). High expression of fibronectin in the tumor bulk stroma was associated to only high T-class (p=0.030). Tenascin-C expression in the tumor bulk stroma associated to cancer survival in the univariate analysis, high expression indicating poor prognosis (log rank p=0.034; Figure 3). In contrast, tenascin-C or fibronectin expression in the tumor invasive front was not associated with clinicopathological variables or cancer survival (Table 2). Multivariate analysis did not show statistically significant associations between tenascin-C or fibronectin expression and survival.

**Table 2:** Expression of tenascin-C and fibronectin in the tumor bulk stroma and invasive front region compared to clinicopathological variables in esophageal adenocarcinoma

Variable	n/N	Tenascin-C bulk, n (%)			Tenascin-C front, n (%)			Fibronectin bulk, n (%)			Fibronectin front, n (%)		
		Low	High	p	Low	High	p	Low	High	p	Low	High	P
pT													
T1	14/89	12 (23)	2 (5)	**0.006**	7 (21)	4 (15)	0.884	6 (13)	8 (19)	**0.030**	8 (22)	3 (12)	0.451
T2	13/89	7 (13)	6 (16)		6 ()	4 (15)		6 (13)	7 (16)		5 (14)	5 (19)	
T3	48/89	30 (58)	18 (49)		18 (55)	17 (63)		31 (67)	17 (40)		19 (53)	17 (65)	
T4	14/89	3 (6)	11 (30)		2 (6)	2 (7)		3 (7)	11 (26)		4 (11)	1 (4)	
Lymph nodes													
negative	35/89	27(52)	8 (22)	**0.004**	19 (58)	10 (37)	0.113	19 (41)	16 (37)	0.693	19 (53)	11 (42)	0.416
positive	54/89	25 (48)	29 (78)		14 (42)	17 (63)		27 (59)	27 (63)		17 (47)	15 (58)	
Organ metastases													
negative	62/89	42 (81)	20 (54)	**0.007**	28 (85)	24 (89)	0.647	32 (70)	30 (70)	0.983	31 (86)	23 (88)	0.785
positive	27/89	10 (19)	17 (46)		5 (15)	3 (11)		14 (30)	13 (30)		5 (14)	3 (12)	
Grade													
Well-differentiated	29/90	21(40)	8 (22)	0.116	15 (45)	8 (30)	0.311	13 (28)	16 (37)	0.623	15 (42)	8 (31)	0.386
Moderately differentiated	23/90	14 (26)	9 (24)		6 (18)	9 (33)		13 (28)	10 (23)		7 (19)	9 (35)	
Poorly differentiated	38/90	18 (34)	20 (54)		12 (36)	10 (37)		21 (45)	17 (40)		14 (39)	9 (35)	
Stage													
I	14/89	11 (21)	3 (8)	**0.038**	8 (24)	4 (15)	0.701	5 (11)	9 (21)	0.491	10 (28)	2 (8)	0.239
II	36/89	24 (46)	12 (32)		17 (52)	16 (59)		21 (46)	15 (35)		18 (50)	15 (58)	
III	12/89	7 (13)	5 (14)		3 (9)	4 (15)		7 (15)	5 (12)		4 (11)	5 (19)	
IV	27/89	10 (19)	17 (46)		5 (15)	3 (11)		13 (28)	14 (33)		4 (11)	4 (15)	
Tumor size													
small (<40mm)	36/88	23 (44)	13 (36)	0.446	12 (36)	12 (44)	0.525	19 (41)	17 (40)	0.937	14 (39)	11 (42)	0.787
large (≥40mm)	52/88	29 (56)	23 (64)		21 (64)	15 (56)		27 (59)	25 (60)		22 (61)	15 (58)	

Significant p-values are shown in bold. High expressions was defined as ≥3.

### Tumor/stroma percentage – relation with clinicopathological variables, cancer survival and correlation with stromal tenascin-C and fibronectin

Both mean and median tumor/stroma percentage in 90 patients with adenocarcinoma were 55% (SD 22). No association to TNM-staging or grade of differentiation was observed whether analyzing in two (cut-off 50%) or four (cut-offs 25, 50, 75%) groups. Similarly, no association to survival was observed (in two groups p=0.983, four groups p=0.869). We observed no correlation with tenascin-C and fibronectin experssion with tumor/stroma percentage (Table 3).

**Table 3:** Correlations of tenascin-C and fibronectin expression with tumor/stroma percentage, α-smooth muscle actin (α-SMA) and vascular density (CD31) with two-tailed Spearman’s correlation test

	Tenascin-C bulk	Tenascin-C front	Fibronectin bulk	Fibronectin front
		Correlation coefficient (p-value)			
Tumor/stroma percentage, n=90	0.096 (p=0.367)	−0.050 (p=0.704)	0.130 (p=0.221)	0.028 (p=0.831)
α-SMA, n=12	0.113 (p=0.727)	−0.111 (p=0.759)	0.118 (p=0.716)	0.211 (p=0.558)
CD31, n=20	0.168 (p=0.465)	−0.357 (p=0.133)	0.175 (p=0.448)	−0.047 (p=0.843)

### Correlation of stromal tenascin-C, fibronectin and α-SMA

**Figure 4:**
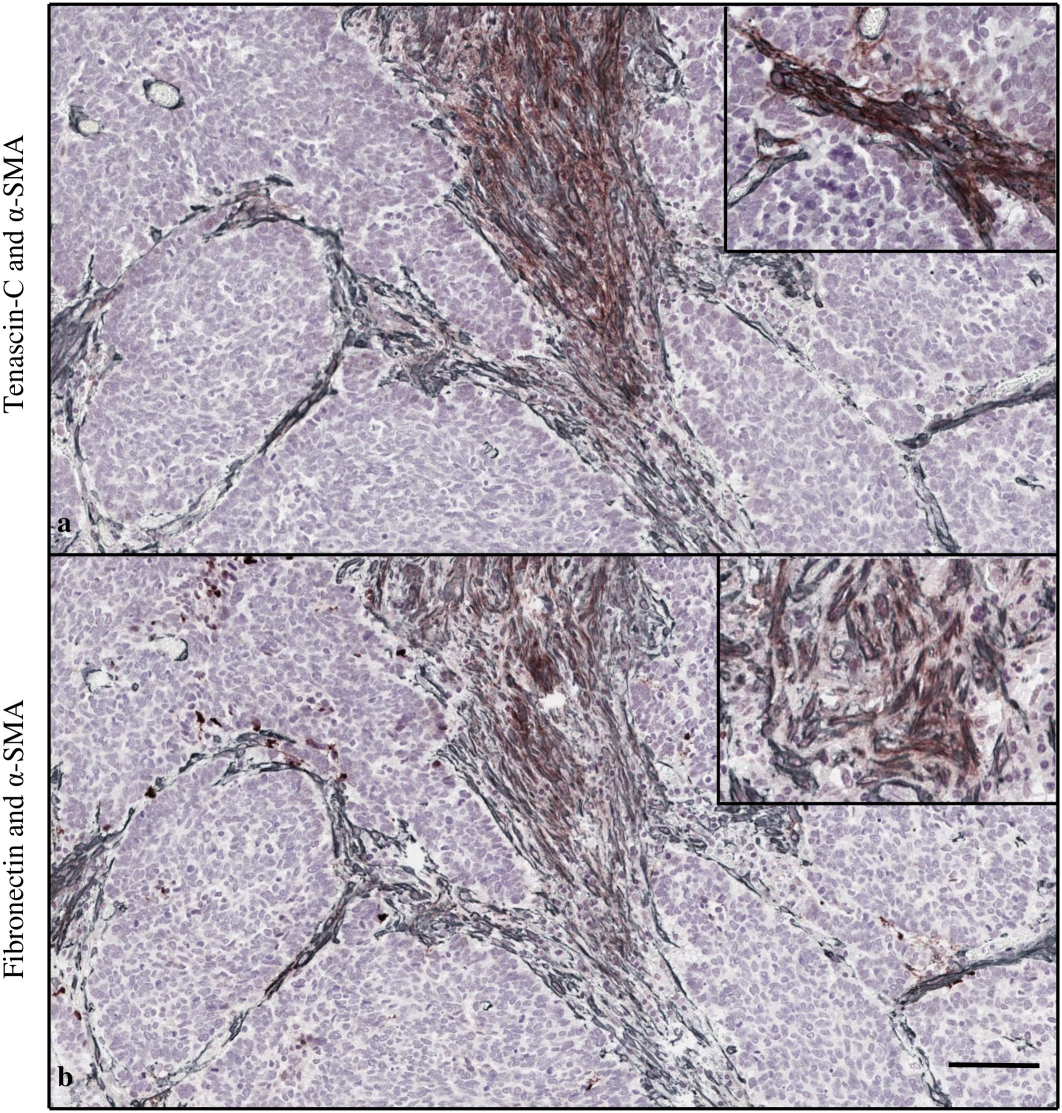
Double staining of tenascin-C (brown) and α-smooth muscle actin (α-SMA, gray) **(a)**. Fibronectin and α-SMA **(b)**. Magnification 10x, scale bar 100 μm. High magnification (20x) images in the right upper corner show stromal cells in the tumor bulk with cytoplasmic positivity of both markers.

Cancer-associated fibroblasts (CAFs), identified by intracellular α-SMA, are known predictors of poor prognosis in various malignancies with highly similar staining pattern as tenascin-C and fibronectin [9, 10]. In 12 selected patient samples, mean α-SMA expression in tumor bulk was 1.9 (SD 1.0) and in tumor invasive front 2.2 (SD 0.8). Overlap with tenascin-C and α-SMA was observed in tumor bulk (26% (SD 32)), and tumor invasive front (13% (SD 15)). Similarly, overlap was seen with fibronectin and α-SMA (42% (SD 46), 56% (SD 44), respectively), Figure 4. Stromal cells in the tumor bulk showed occasional cytoplasmic positivity of both markers (Figure 4). However, no statistically significant correlation in tenascin-C or fibronectin expression and α-SMA was observed (Table 3).

### Correlation of stromal tenascin-C, fibronectin and vascular density

CD31 staining for analysis of mean microvascular density was performed to 20 randomly selected patients. Mean number of vessels per single high power field (20x magnification) in tumor bulk was 48 (SD 20) and in tumor invasive front 56 (SD 33). We observed no correlation with tenascin-C or fibronectin expression and microvessel density (Table 3).

## DISCUSSION

We demonstrate the expression of tenascin-C and fibronectin in normal esophageal mucosa and in columnar metaplasia-dysplasia-adenocarcinoma-sequence of Barrett’s esophagus. Stromal expression of both of the molecules showed stepwise increase from metaplasia towards esophageal adenocarcinoma. Tenascin-C expression was generally more abundant than fibronectin and high Tenascin-C expression in the tumor stroma significantly associated with advanced TNM-stage and poor prognosis. High fibronectin expression was associated with advanced T-stage.

Strength of the current study is homogenous study population from single geographical area of Northern Finland from 1987 to 2013 with no apparent selection bias. Interobserver agreement was excellent with no required re-evaluations. Possible weakness is the use of immunohistochemistry as the only method in the evaluation of tenascin-C and fibronectin expression. However, strict validation of the used antibodies was performed and the inverse association with tenascin-C expression and survival with solid biological background in cancer metabolism reduces the possibility of chance findings.

Based on our results, tenascin-C expression is upregulated in stroma of esophageal adenocarcinoma when compared to gastric and intestinal metaplasia and low- and high-grade dysplasia. Several previous studies have demonstrated that the high tenascin-C expression in the tumor microenvironment is caused by its increased synthesis in myofibroblasts [11]. It has also been reported that cancer cells in micrometastases act as the main source of tenascin-C in the tumor until the tumor stroma can take over the tenascin-C production [12]. By expressing tenascin-C, the cancer cells may support their own ability to metastasize. In our study, intracellular staining for tenascin-C in the cancer cells as a possible indicator of synthesis was rare and no association with clinicopathological features was seen. In oral tongue squamous cell carcinoma, a similar staining pattern of CAFs with tenascin-C, fibronectin has been previously observed [10, 13]. With a pilot series of 12 patients we observed overlap of tenascin-C, fibronectin and α-SMA expressions in tumor stroma and in cytoplasm of stromal cells suggesting that myofibroblasts contribute to stromal tenascin-C and fibronectin synthesis. In agreement with our results, tenascin-C expression has usually been observed in the tumor stroma [14]. Its distribution is determined primarily by location of the cells that secrete it, but it is probable that its binding to specific proteins, for example collagens, is also involved [15].

Previous studies have suggested that tenascin-C could be used as a biomarker of tumor invasion and metastasis and as a prognostic factor in various cancers [3, 4]. It has also been investigated as a target for therapy [16]. In colorectal cancer myofibroblasts in stroma of invasive tumor front secrete tenascin-C and stimulate cancer cell invasion [11, 17]. In our study, tenascin-C and fibronectin expression were significantly increased in the tumor invasive front as compared to the tumor bulk stroma without showing any association with clinicopathological variables. In contrast, the expression of tenascin-C in the tumor bulk of esophageal adenocarcinoma tumors was related to higher TNM-stage. The mechanisms for the association of abundant tenascin-C with adverse cancer prognosis may lie in its various mechanisms affecting both the structure of extracellular matrix and functions of cancer cells. Previously, high tenascin-C expression has been shown to associate to poor cellular differentiation, although with limited patient material [5]. Tenascin-C is able to influence cancer growth by affecting cell adhesion and migration, but also by influencing the expression of tumor suppressor genes, oncogenes and genes involved in the maintenance of genomic stability [18, 19]. It is also known to have a role in loss of intercellular adhesion [20], enhancing cell migration and metastasis of tumor cells. In our study, no correlation with vascular density and tenascin-C or fibronectin expression was observed, suggesting that prognostic effect of tenascin-C is not related with induction of angiogenesis. Why expression patterns only in the tumor bulk stroma and not in the invasive front were associated to poor prognosis, is unclear. Based on largely constant abundant expression of tenascin-C in the invasive front (Table 1), we speculate, that the expression in the invasive front might be upregulated by the process of invasion itself, while expression patterns in the tumor bulk might be more representative of those properties of tumor cells important in the distant disseminaton.

Fibronectin showed a similar pattern of upregulation as tenascin-C in adenocarcinoma samples. Expression in normal mucosa, both types of metaplasia and dysplasia was more or less the same and only adenocarcinoma differed significantly from all other lesion types. Fibronectin controls a variety of physiological functions, including cell adhesion, cell migration, cell differentiation, tissue repair and cell proliferation [8]. Fibronectin stimulates cell growth and reduces apoptosis through integrin α_5_β_1_ activation, cyclooxygenase-2 induction and by inhibiting cyclin-dependent kinase inhibitor p21^Waf1/Cip1^ gene expression [21]. It also activates several kinase-signaling pathways of which are not shared by other matrix components [6]. It has been earlier shown that the adhesion of lung carcinoma cells to fibronectin reduces apoptosis induced by standard chemotherapeutic agents and enhances tumorigenicity [22], which supports the hypothesis that fibronectin could have value as a therapeutic target in cancers [23]. Our results indicate that fibronectin expression increases with the progression of esophageal adenocarcinoma and that its high expression associates to T-stage of the tumor. In agreement with several previous studies, it seems that fibronectin is related to cancer development and growth.

In our study tumor/stroma percentage showed no association to clinicopathological variables or survival. Previously one study in esophageal adenocarcinoma has suggested that low (<50%) percentage indicates poor prognosis [24]. On the contrary, in colorectal cancer high tumor/stroma percentage was associated to a reduced cancer-specific survival [25]. More studies are needed to assess the significance of tumor/stroma percentage in esophageal cancer. Interestingly, we observed no correlation with tumor/stroma percentage and tenascin-C or fibronectin expression, suggesting that the size of stromal area does not affect tenascin-C or fibronectin density.

In conclusion, our results indicate that both tenascin-C and fibronectin are highly expressed in the stroma of esophageal adenocarcinoma and that expression in the precursor stages, including Barrett’s esophagus and dysplasia, is low compared to the cancer tissue. In addition, the results show that high tenascin-C expression in tumor stroma associates with advanced disease and reduced survival, and could therefore be used as a biomarker for esophageal adenocarcinoma with poor prognosis.

## MATERIALS AND METHODS

### Patients

Paraffin-embedded archival specimens of adenocarcinoma or esophageal dysplasia were collected from the Department of Pathology, Oulu University Hospital, between years 1987-2013, with follow-up until the end of 2015 or death. The final series consisted of 90 patients with esophageal adenocarcinoma, 10 with high-grade dysplasia and 20 with low-grade dysplasia as the most advanced lesion. Of the carcinoma specimens, 26 (29%) were biopsies and 64 (71%) surgical specimens. The material has been previously described elsewhere [26–28]. Out of 90 patients with carcinoma, four received neoadjuvant chemoradiotherapy. The median age of the patients with adenocarcinoma was 64 years (range 43-91). The median follow-up time was 38 months (range 0-183). The data for the patient survival was obtained from Statistics Finland, and other relevant data was obtained from the patient records (Table 4).

**Table 4:** Baseline characteristics of the patients with esophageal adenocarcinoma, high grade dysplasia and low-grade dysplasia

Patient clinical data		EAC N=90		HGD N=10		LGD N=20	
Age at diagnosis		n/N	%	n/N	%	n/N	%
	<60 yrs	32/90	35.6	5/10	50	4/20	20
	60-65yrs	18/90	20.0	3/10	30	3/20	15
	>65 yrs	40/90	44.4	2/10	20	13/20	65
**Sex**							
	Male	74/90	82.2	10/10	100	13/20	65
	Female	16/90	17.8	0/10	0	7/20	35
**Tumor grade**							
	Well-differentiated	29/90	32.2				
	Moderately differentiated	23/90	25.6				
	Poorly differentiated	38/90	42.2				
**T-classification**							
	I	14/89	15.7				
	II	13/89	14.6				
	III	48/89	53.9				
	IV	14/89	15.7				
**Lymph nodes**							
	Negative	35/89	39.3				
	Positive	54/89	60.7				
**Distant metastases**							
	Negative	62/89	69.7				
	Positive	27/89	30.3				
**Tumor stage**							
	I	14/89	15.7				
	II	36/89	40.4				
	III	12/89	13.5				
	IV	27/89	30.3				

Total of 90 patients with adenocarcinoma, 10 patients with high-grade dysplasia and 20 patients with low-grade dysplasia are presented in the Table. TNM classification was available only from 89 patients.

The use of patient samples and the data inquiry were approved by the Oulu University Hospital Ethics Committee. The need to obtain a written or oral consent from the patients for using the samples in research was waived by the Finnish National Authority for Medicolegal Affairs (VALVIRA, Dnro 10832/06.01.03.01/2014).

### Immunohistochemistry

The representative tissue blocks for immunohistochemistry were selected by an expert gastrointestinal pathologist on the basis of hematoxylin and eosin-stained sections, based on presence of cytological abnormalities described in detail previously [29, 30]. Sections of 5μm were mounted and dewaxed in xylene. For antigen retrieval, the sections were heated in Tris-EDTA solution (pH 9) for 10 min. Endogenous peroxidase activity was blocked using peroxidase-blocking solution (DAKO, Copenhagen, Denmark) for 10 min. Incubation with primary antibodies D2 (Tenascin-C) and F12 (Fibronectin) in dilution of 1:500 for 60 min at room temperature was done. Previously described and validated antibodies against Fibronectin and Tenascin C were used [13]. The antibodies were validated by comparison to commercially available fibronectin and tenascin-C antibodies, as well as using mass spectrometry. Horseradish peroxidase (HRP)-conjugated antibodies (DAKO) were introduced to sections for 10 min, after which DAB chromogen was used for 5 minutes. Slides were counterstained with haematoxylin. We validated the immunohistochemical analysis through two series of negative controls (omitting the primary antibody and by replacing primary antibody with the mouse primary antibody isotype control).

α-smooth muscle actin (α-SMA) staining with primary antibody (Dako monoclonal mouse anti-human, 1:500, clone 1A4, M0851) for 30min in room temperature was performed to 6 patient samples with high (≥3) tenascin-C and fibronectin expression, and 6 samples with low tenascin-C and fibronectin expression. Double-stains of α-SMA, tenascin-C and fibronectin were performed by EnVision G|2 Doublestain kit (Dako) according to manufacturer’s instructions.

### Assessment of tenascin-C and fibronectin expression

The hematoxylin and eosin-stained histological samples were digitalized using Aperio AT2 Console, Leica Biosystems Imaging Inc, Nussloch, Germany. Identification and marking of the different lesions in the samples were made by an expert gastrointestinal pathologist (T.J.K). Immunoreactivity of tenascin-C and fibronectin were analyzed by two independent researchers who were blinded from the clinical data. Non-epithelial (stromal) parts and epithelial cells were separately assessed. We analyzed the amount and pattern of tenascin-C and fibronectin expression in the stroma with a 5 point scale slightly modified from that described earlier [10]; 0, no detectable staining; 1, focal staining; 2, areas diffuse staining present in less than half of stromal area; 3, expression of moderate density distributed in more than half but not in all parts of the tumor stroma; 4, dense expression extending throughout the stroma. The intensity of staining in the epithelial cells was estimated on a four point scale (0=negative, 1=weak, 2=moderate, 3=strong). We also estimated the percentage of the stained epithelial cells (0-100%). If there was no difference over 1 in the intensity or over 30% in the percentage between the two independent estimations, the mean values were used in the study. If the difference was greater, re-evaluation was performed with a third researcher. However, in the current study re-evaluation was not needed in any of the samples.

With the cancer lesions we estimated separately the stromal expression in tumor bulk and in the invasive front of the tumor. The entire area of the target lesion was screened and expression was analyzed from area of predominant staining. In biopsy samples the invasive front of the tumor could not be reliably identified, and so the comparison between the tumor stroma and front was limited to the resected adenocarcinoma samples (n=64).

### Assessment of tumor/stroma percentage

Tumor/stroma percentage was evaluated from hematoxylin and eosin-stained sections from all carcinoma patients (n=90) as described earlier [24]. Percentage (0-100%) of tumor area in representative microscope view from invasive margin was assessed, referred to as tumor/stroma percentage. Mean value of two independent researchers were used with no need for re-evaluation, as the interobserver difference was less than 30%. Significance of tumor/stroma percentage was analyzed by using two (≤50% and >50%) and four (0-25%, 26-50%, 51-75% and 76-100%) groups.

### Assessment of double stains of α-smooth muscle actin, tenascin-C and fibronectin

Double stained samples were digitalized similarly as described above. Expression of α-SMA was analysed in the tumor bulk and in the invasive front of the tumor with 5-point scale similarly as tenascin-C and fibronectin. The overlap of tenascin-C and α-SMA staining was assessed in both tumor bulk and invasive front. The overlap was reported as percentage (0-100%) of tenascin-C positive stroma which contained also α-SMA positivity. Fibronectin and α-SMA were analysed with similar manner. Mean value of two independent researchers were used with no need for re-evaluation.

### Immunostaining for CD31 and assessment of vascular density

To study correlation of vascular density, tenascin-C and fibronectin expression we randomly selected 20 samples for staining with an antibody to CD31 (M0823, clone JC70A, Dakopatts, Glostrup, Denmark) with a dilution of 1:200. The slides were counterstained with hematoxylin. The mean vascular density was calculated as the mean number of positively stained blood vessels in high power (20x magnification) field. Any endothelial cell cluster consisting of two or more cells was considered a single, countable microvessel. Minimum of 10 high power fields were analyzed from each tumor and the number of microvessels was divided by the number of fields assessed to obtain mean vascular density.

### Statistical analysis

For statistical analysis we used IBM SPSS 22.0 (IBM corp., Armonk, NY, USA). The evaluated mean values of tenascin-C and fibronectin were divided into two different categories (low and high ≥3 expression). Due to only marginally skewed distributions, we used Oneway ANOVA with Tukey in post hoc analysis to compare tenascin-C and fibronectin expressions between the different lesions. Comparison between tumor stroma and invasive front was performed with paired sample T-test. The chi-square test was used to calculate statistically significant differences between the protein expression and clinicopathological variables. Life tables were calculated with the Kaplan-Meier method and compared between low and high (≥3) tenascin-C and fibronectin stromal expression density, and tumor/stroma percentage (≤50% and >50% or 0-25%, 26-50%, 51-75% and 76-100%). Statistical significance was assessed with log-rank test. Multivariable analysis was done using Cox proportional hazards model with the following covariates: T- (1-4), N- (negative or positive), M-stage (negative or positive) and tenascin-C expression in tumor bulk (low or high; (≥3), using backward stepwise selection of variables. P-value of 0.05 was used as the limit for inclusion of a covariate. Backward stepwise algorithm was used to pick the best combination of prognostic factors to explain the mortality in the study population. Correlation between stromal tenascin-C, fibronectin, tumor/stroma percentage, α-SMA and CD31 were calculated using two-tailed Spearman’s correlation test.
